# Supplementary material for: Myelin damage in donor skin differentiates between synucleinopathies
Source: NPJ Parkinsons Dis. 2026 May 19;12:186. doi: 10.1038/s41531-026-01385-w (PMC13447855; doi:10.1038/s41531-026-01385-w)
Supplement: Supplementary file 1 [file 41531_2026_1385_MOESM1_ESM.pdf]

Supplementary Material

	CTRL	PD	DLB	MSA
**Number**	11	23	6	6
**Sex (F/M)**	7/4	6/17	1/5	3/3
**Age at death[a] (years ± SD)**	79.9 ± 9.2	76.9 ± 6.1	77.0 ± 3.6	60.0 ± 7.3
**Age at death[b] (years ± SD)**	79.9 ± 9.2	77.0 ± 6.1	77.3 ± 2.9	61.2 ± 7.1
**Age at onset (years ± SD)**	na	60.4 ± 10.2	70.2 ± 5.3	53.7 ± 8.9
**Disease duration (years ± SD)**	na	16.4 ± 7.6	7.2 ± 3.8	7.3 ± 5.6
**PMD[a] (h)**	4.7 ± 1.5	4.2 ± 1.2	5.0 ± 2.5	5.5 ± 2.4
**PMD[b] (h)**	4.7 ± 1.5	4.9 ± 2.0	5.9 ± 2.4	5.4 ± 2.2
**Fixation time[a] (h)**	33 ± 23	29 ± 10	24 ± 1	44 ± 22
**Thal amyloid phase**	0-4 (4/4/0/2/1*)	0-4 (5/8/6/3/1)	0-5 (1/0/0/3/1/1)	0-4 (3/1/0/0/2)
**Braak NFT stage**	0-3 (1/3/1/6)	0-3 (5/5/8/5)	2-4 (1/3/2)	0-4 (3/1/0/1/1)
**Braak αSyn stage**	0-6 (9/1/0/0/0/0/1**)	5-6 (1/22)	6 (6)	0-5 (2/0/1/0/0/3)

Supplementary Table 1: Clinicopathological characteristics of donors included in this study. PMD: post-mortem delay, NFT: neuro-fibrillary tangle, LB: Lewy body, [a]: donors for EM studies, [b]: full cohort. Brackets indicate the number of donors within each stage. *Non-neurological control with age-associated pathology (#Case 3). **Non-neurological control with age-associated pathology (incidental LB) (#Case 2).

Name	ID #	Company	Target	Tissue - Brain		Tissue - Skin	
				AR	Condition	AR	Condition
MPZ – P0	ab183868	Abcam	MPZ – aa 1-258	-	-	yes[a]	o/n, 4°C, 1:100
LB509	180215	Thermofisher	αSyn - aa 115-122	no	1h, 37°C, 1:500	yes[b]	o/n, 4°C, 1:100
11A5	-	Prothena	αSyn - pS129	no	1h, 37°C, 1:100000	yes[b]	o/n, 4°C, 1:100
αSyn clone EP1536Y	ab51253	Abcam	αSyn - pS129	no	1h, 37°C, 1:5000	yes[b]	o/n, 4°C, 1:100
αSyn C terminal	A15127A	Biolegend	αSyn - CTT 122	yes[b]	o/n, 4°C, 1:500	yes[b]	o/n, 4°C, 1:100
αSyn N terminal	A15110D	Biolegend	αSyn - aa 34-45	yes[b]	o/n, 4°C, 1:500	yes[b]	o/n, 4°C, 1:100
131 Roche	-	Roche	αSyn - CTT 119	no	1h, 37°C, 1:500	yes[b]	o/n, 4°C, 1:100
134 Roche	-	Roche	αSyn - CTT 122	no	1h, 37°C, 1:500	yes[b]	o/n, 4°C, 1:100
142 Roche	-	Roche	αSyn - pS129	no	1h, 37°C, 1:500	yes[b]	o/n, 4°C, 1:100

Supplementary Table 2: Primary antibodies used in this study for immunohistochemistry on 200 nm resin sections. [a] :10’ in formic acid at RT + 30’ at 95 °C in Tris-EDTA pH9. [b] :10’ in formic acid at RT + 30’ at 95 °C in sodium citrate pH6 or 10’ in formic acid at RT + 30’ at 95 °C in Tris-EDTA pH9. AR: antigen retrieval, MPZ: myelin protein zero, aa: amino acids, CTT: C-terminal truncated, o/n: overnight, RT: room temperature.

ID	Study	Clinical Diagnosis	Pathological diagnosis	Onset Age	Disease duration	Gender	Cause of death
Case 1	**EM**	Control	Control	NaN	NaN	F	Euthanasia, breast cancer
Case 2*	**EM, ELISA**	Control	Control with age-associated pathology (iLBD)	NaN	NaN	F	Euthanasia, cancer
Case 3	**EM**	Control	Control with age-associated pathology	NaN	NaN	M	Euthanasia, rectal carcinoma
Case 4	**EM, FFPE**	Control	Control	NaN	NaN	M	Euthanasia, metastasis prostate carcinoma, spinal cord metastasis
Case 5	**EM**	Control	Control	NaN	NaN	F	Euthanasia, pancreatic carcinoma
Case 6	**EM, ELISA, FFPE**	Control	Control	NaN	NaN	M	Euthanasia
Case 7	**EM, ELISA**	Control	Control	NaN	NaN	F	Heart attack
Case 8	**EM, ELISA**	Control	Control	NaN	NaN	F	Heart failure
Case 9	**EM, ELISA**	Control	Control	NaN	NaN	M	Euthanasia, bile duct cancer, intraheptic cholangiocarcinoma with lymph node and peritoneal metastases
Case 10	**EM, ELISA**	Control	Control	NaN	NaN	F	Ceased oral intake
Case 11	**EM, ELISA, FFPE**	Control	Control	NaN	NaN	F	Euthanasia, achalasia of the esophagus
Case 12	**EM, ELISA**	PD	PD	NaN	NaN	M	Euthanasia
Case 13	**EM, ELISA**	PD	PD	51	22	M	Euthanasia
Case 14	**EM**	PD	PD	65	20	M	Palliative sedation, prostate cancer
Case 15	**EM**	PD	PD	NaN	NaN	F	Euthanasia
Case 16	**EM, ELISA**	PD	PD	62	16	M	Cardiac failure
Case 17	**EM, ELISA**	PD	PD	69	18	M	Euthanasia
Case 18	**EM**	PD	PD	69	14	M	Dehydration
Case 19	**EM, ELISA, FFPE**	PD	PD	45	28	F	Euthanasia
Case 20	**EM, ELISA**	PD	PD	43	21	F	Euthanasia
Case 21	**EM, ELISA**	PD	PD	48	24	M	Euthanasia
Case 22	**EM**	PD	PD	69	6	M	Aspiration/pneumonia
Case 23	**EM**	PD	PD	64	10	M	Euthanasia
Case 24	**EM, ELISA**	PD	PD	77	4	F	Euthanasia
Case 25	**EM, ELISA**	PD	PD	63	11	F	Cachexia, end stage disease
Case 26	**EM, ELISA**	PD	PD	62	11	F	Pneumonia, terminal stage
Case 27	**EM, ELISA**	PD	PD	52	30	M	Euthanasia
Case 28	**ELISA**	PD	PD	68	10	M	Dehydration, end stage disease
Case 29	**ELISA**	PD	PD	64	14	M	Natural death, dehydration
Case 30	**ELISA, FFPE**	PD	PD	70	7	M	Euthanasia
Case 31	**ELISA, FFPE**	PD	PD	73	11	M	Euthanasia
Case 32	**ELISA**	PD	PD	42	22	M	Palliative sedation, cancer
Case 33	**ELISA**	PD	PD	55	29	M	Fall out of bed
Case 34	**ELISA**	PD	PD	58	16	M	Euthanasia
Case 35	**EM, ELISA**	DLB	DLB	73	6	M	Fever, infection
Case 36	**EM, ELISA**	DLB	DLB	64	10	M	Euthanasia
Case 37	**EM**	DLB	DLB	78	3	M	natural death
Case 38	**EM, ELISA, FFPE**	DLB	DLB	67	7	M	Dehydration, end stage disease
Case 39	**ELISA, FFPE**	DLB	DLB	66	13	M	Palliative sedation, end stage dementia
Case 40	**ELISA, FFPE**	DLB	DLB	73	4	F	Palliative sedation, end stage disease
Case 41	**EM, ELISA, FFPE**	MSA	MSA	63	4	F	Euthanasia
Case 42	**EM, ELISA**	MSA	MSA	45	6	F	Euthanasia
Case 43	**EM, ELISA, FFPE**	MSA	MSA	46	8	M	Coma in last few days (collapse ventilation stopped)
Case 44	**EM**	MSA	MSA	66	2	F	Deceased in sleep
Case 45	**EM, ELISA**	MSA	MSA	53	6	M	Euthanasia
Case 46	FFPE	MSA	MSA	49	18	M	Pneumonia, sepsis

ID	Age	PMD (h)	Fixation time (h) EM samples only	Thal	NFT	Braak (LB)	Neuropath. score	ARTAG	CAA	LEDD/day (mg)
Case 1	57	7.00	24.00	1	0	0	A1B0C0			0
Case 2*	86	5.75	24.00	1	3	6	A1B2C0			0
Case 3	81	4.42	22.58	4	3	0	A3B2C2			0
Case 4	77	4.00	89.00	1	3	0	A1B2C0			0
Case 5	79	2.75	18.00	3	3	0	A2B2C0			0
Case 6	76	3.50	24.00	0	1	0	A0B1C0		Type 2 stage 1	0
Case 7	82	7.00	34.50	0	1	0	A0B1C0			0
Case 8	80	3.83	65.25	0	1	0	A0B1C0	yes		0
Case 9	79	6.00	19.25	1	3	0	A1B2C0	yes		0
Case 10	93	3.50	24.00	3	3	0	A2B2C0	yes		0
Case 11	89	3.75	23.50	0	2	1	A0B1C0	yes		0
Case 12	83	2.92	20.00	1	3	6	A1B2C0			NaN
Case 13	73	4.83	24.67	1	3	6	A1B2C0	yes, amygdala	Type 2, slight	1250
Case 14	85	4.75	25.00	1	2	6	A1B1C0	yes, olive	Type 2, slight	690
Case 15	74	6.75	23.67	1	1	6	A1B1C0	yes, amygdala		NaN
Case 16	78	5.42	28.50	2	2	5	NaN			600
Case 17	87	3.83	22.83	3	2	6	A2B1C0	yes, amygdala slight	Type 2, slight	480
Case 18	83	3.50	NaN	1	1	6	NaN	very severe		900
Case 19	73	3.58	24.00	0	0	6	A0B0C0			475
Case 20	64	1.83	24.00	4	3	6	A3B2C1		Type 2, slight	938
Case 21	72	3.67	24.00	2	1	6	A1B1C0		Type 1, slight	1088
Case 22	75	4.67	37.25	3	2	6	A2B1C1		Type 2	600
Case 23	74	3.67	24.00	2	2	6	A2B1C0			1400
Case 24	81	4.50	24.00	2	0	6	A1B0C0			300
Case 25	74	4.75	38.75	3	3	6	A3B2C0	yes		400
Case 26	73	5.25	28.25	1	3	6	A1B2C0	yes		NaN
Case 27	82	3.00	60.67	0	0	6	A0B0C0	yes		100
Case 28	78	7.75	NaN	1	2	6	A1B1C0			NaN
Case 29	78	10.25	NaN	2	2	6	NaN			NaN
Case 30	77	4.33	NaN	2	1	6	A1B1C0		Type 1, slight	NaN
Case 31	84	4.67	NaN	0	2	6	A0B1C0	yes		NaN
Case 32	64	4.42	NaN	0	0	6	A0B0C0			NaN
Case 33	84	9.58	NaN	1	0	6	A1B0C0	yes	Type 2, slight	1200
Case 34	74	4.25	NaN	0	1	6	A0B1C0	yes		100
Case 35	79	3.75	24.25	3	3	6	A2B2C1	yes		150
Case 36	74	4.00	21.75	4	4	6	A3B2C1	yes		450
Case 37	81	8.75	24.00	5	4	6	A3B2C2	yes	moderate	NaN
Case 38	74	3.67	24.50	3	3	6	A2B2C0			0
Case 39	79	8.33	NaN	0	2	6	A0B1C0		Type 2, moderate	NaN
Case 40	77	6.75	NaN	3	3	6	A2B2C3	yes		NaN
Case 41	67	4.33	75.00	4	3	5	A3B2C1			1918
Case 42	52	4.67	36.00	1	1	5	A1B1C0		Type 2, slight	2168
Case 43	54	4.42	57.58	0	0	5	A0B0C0	yes		NaN
Case 44	68	9.83	24.17	0	0	2	A0B0C0			0
Case 45	59	4.17	24.83	0	0	0	A0B0C0			0
Case 46	67	5.25	NaN	4	4	0	NaN			NaN

ID	Hallucinations	Cognitive impairment	Cognitive value (CDR)	Urinary tract infection	Sleep problems	Hypotension	Constipation
Case 1	NaN	NaN	NaN	NaN	NaN	NaN	NaN
Case 2*	NaN	NaN	NaN	NaN	NaN	NaN	NaN
Case 3	NaN	NaN	NaN	NaN	NaN	NaN	NaN
Case 4	NaN	NaN	NaN	NaN	NaN	NaN	NaN
Case 5	absent	no	0	absent	absent	absent	absent
Case 6	NaN	NaN	NaN	NaN	NaN	NaN	NaN
Case 7	absent	no	0	absent	absent	absent	absent
Case 8	NaN	NaN	NaN	NaN	NaN	NaN	NaN
Case 9	NaN	NaN	NaN	NaN	NaN	NaN	NaN
Case 10	absent	no	0	absent	absent	absent	absent
Case 11	absent	no	0	absent	absent	absent	absent
Case 12	NaN	NaN	NaN	NaN	NaN	NaN	NaN
Case 13	present	mild	1	absent	absent	present	present
Case 14	absent	no	0	present	absent	present	present
Case 15	NaN	NaN	NaN	NaN	NaN	NaN	NaN
Case 16	present	impaired	2	present	absent	absent	present
Case 17	present	no	0	absent	present	present	present
Case 18	present	impaired	2	present	present	absent	present
Case 19	present	no	0	absent	present	absent	present
Case 20	present	no	0	present	present	absent	present
Case 21	present	mild	1	present	absent	absent	absent
Case 22	present	severe	3	present	present	present	present
Case 23	present	mild	1	present	present	absent	present
Case 24	absent	no	0	absent	absent	absent	present
Case 25	present	severe	3	present	absent	present	absent
Case 26	present	impaired	2	present	absent	present	absent
Case 27	present	no	0	absent	present	present	absent
Case 28	absent	mild	1	absent	present	present	present
Case 29	present	advanced	3	absent	present	present	absent
Case 30	absent	no	0	absent	present	absent	absent
Case 31	absent	no	0	absent	present	absent	present
Case 32	present	mild	1	present	present	absent	present
Case 33	present	yes but not specified	NaN	absent	present	absent	present
Case 34	absent	no	0	absent	present	NaN	present
Case 35	present	severe	3	absent	absent	present	absent
Case 36	present	severe	3	absent	present	present	absent
Case 37	NaN	NaN	NaN	NaN	NaN	NaN	NaN
Case 38	present	impaired	2	absent	present	absent	absent
Case 39	present	Severe	3	present	present	present	absent
Case 40	present	advanced	3	absent	present	present	absent
Case 41	absent	mild	1	absent	absent	absent	present
Case 42	absent	no	0	present	present	present	present
Case 43	absent	no	0	absent	present	present	present
Case 44	absent	no	0	present	present	present	present
Case 45	absent	mild	1	present	present	absent	present
Case 46	present	impaired	2	absent	present	present	absent

Supplementary Table 3. Full clinico-pathological report of each donor used in this study. EM: electron microscopy; ELISA: Enzyme-linked immunosorbent Assay; PD: Parkinson's disease; DLB: Dementia with Lewy bodies; MSA: multiple system atrophy; iLBD: incidental Lewy body disorder; NaN: not available / not applicable. F/M: female/male; PMD: post-mortem delay; h: hours; Thal: Thal amyloid phase; Braak NFT: neuro-fibrillary tangle; Braak (LB): Braak αSyn stage (Lewy body); Neuropath. score: Neuropathological score; ABC score: score for Alzheimer's disease and neuropathological change[1,2]. A: Amyloid-beta deposits, B - neuro-fibrillary tangle, C: neuritic plaques; ARTAG; ageing related tau astro-gliopathy; CAA: cerbral amyloid angiopathy; LEDD (mg): Levadopa equivalent daily dose/milligram; CDR: clinical dementia rating. Many clinical symptoms reported in this table such as the presence of cognitive impairment, hallucinations, urinary tract infections, sleep problems, constipation, hypotension were not uniformly, systematically and longitudinally assessed throughout the cohort, therefore serve solely as a qualitative indication of the general condition of the donors, rather than a medical assessment.*Note: this case had very few LBs and LNs in the brainstem, medial temporal cortex, and few in the cingulate cortex. The Frontal and parietal cortex were negative, however motor cortex was positive, with few inclusions. This fits with iLBD, as the number of inclusions in the limbic and cortical regions is limited. 35% of the myelin sheaths analysed for this donor exhibited no damage, 38% mild damage, 27% moderate damage and

0% high damage, in between the CTRL and PD/DLB categories, with respect to the proportion of damaged myelin sheaths (Figure 3, panel a). The total myelin damage score for this donor was 0.92, therefore still considered as a control being <1. Interestingly, the clinical file reported "bradykinesia left arm/hand, no tremor. Differential diagnosis possible underlying PD with depressive symptoms. Unclear presentation of bodily symptoms".

	**Drug**	**Dosage (mg)**	**Purpose**	**Note**
**Phase 1**	Thiopental	2000	Induce loss of consciousness	Can cause depression of autonomic ganglia and local tissue injury due to its ischemic chemical properties. It only has local effects and the skin biopsies used in this study were collected at a distant site (C6-T1) compared to the injection site (intravenous route).
	Propofol	1000		Acts as a potent pain-inducing agent upon injection, but also exhibiting potential analgesic, anti-inflammatory and neuroprotective properties when administered locally[3]. Can induce toxicity to developing nervous system[4], however the skin biopsies were collected in an aged cohort (52-93 years old) for this study.
**Phase 2**	Rocuronium	150	Relax muscles and induce stop in breathing	Non-depolarizing neuromuscular blocking agents that act on the peripheral nervous system by competitively binding to nicotinic acetylcholine receptors (nAChR) at the postjunctional membrane of the neuromuscular junction. They inhibit nerve-induced muscle contractions, resulting in skeletal muscle paralysis with a local effect without creating peripheral nerve injuries[5,6].
	Atracurium	100		
	Cisatracurium	30		

Supplementary Table 4: The euthanasia procedure is done according to the national federation of medical doctors in The Netherlands (Royal Dutch Medical Association - KNMG). At the time of the euthanasia, the donors are given one of the two drugs in "Phase 1" to induce loss of consciousness and later receive one of the three drugs in "Phase 2" to relax muscles and induce stop in breathing. According to the mechanism of action of the drugs used for the euthanasia, we believe that this procedure is not responsible for the observed myelin damages.

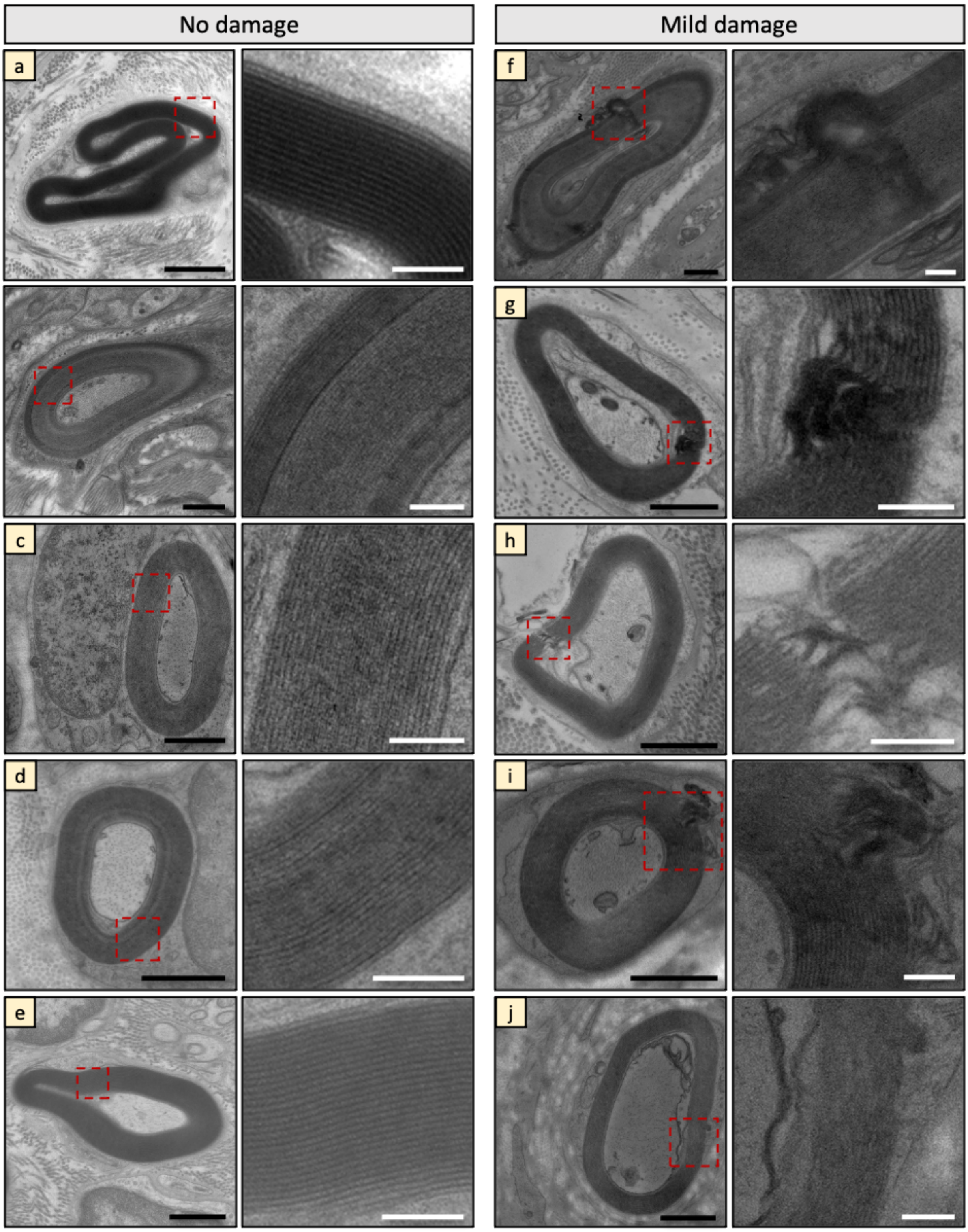

Supplementary Figure 1: EM images of dermal myelin sheaths (no and mild damage categories). Examples of non-damaged (a-e) and mildly damaged (f-j) myelinated axons with their zooms (dashed red square). Scale bars: low magnification 1 µm, high magnification 200 nm.

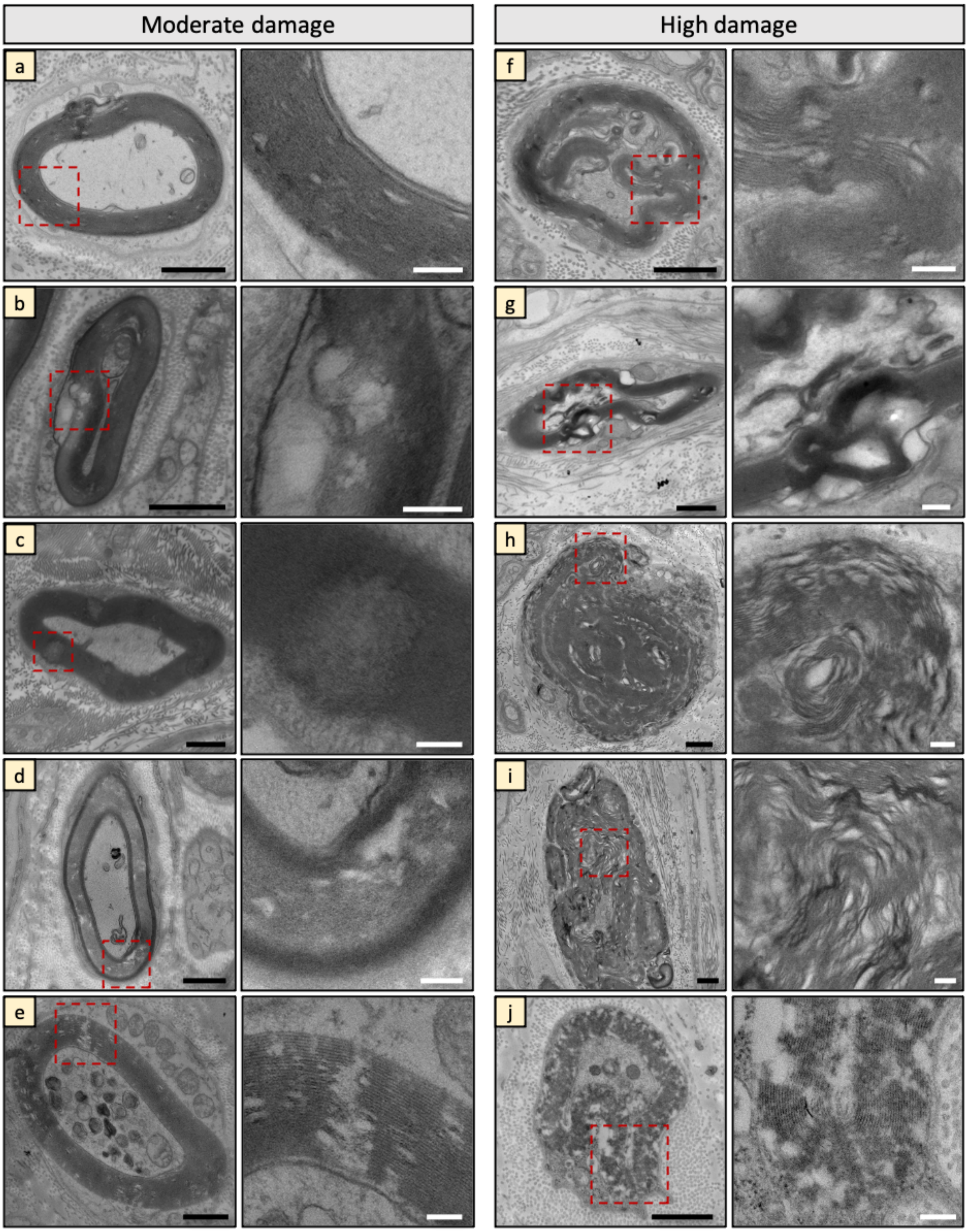

Supplementary Figure 2: EM images of dermal myelin sheaths (moderate and high damage categories). Examples of moderately (a-e) and highly damaged (f-j) myelinated axons with their zooms (dashed red square). Scale bars: low magnification 1 µm, high magnification 200 nm.

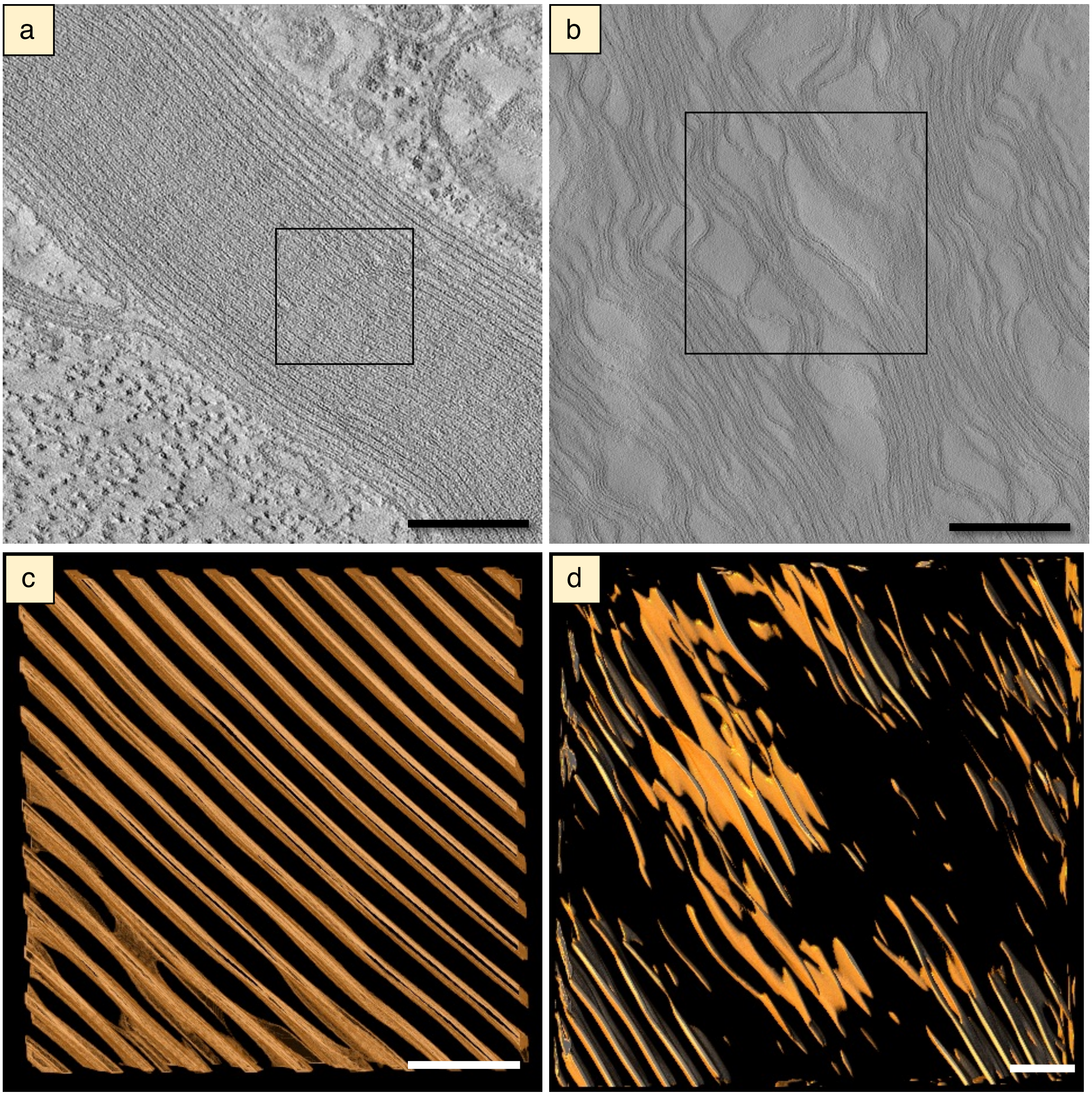

Supplementary Figure 3: Examples of electron tomography and segmentation of no and high myelin damage categories. EM tomography from the preserved and highly damaged categories of myelin damage. (a) Tomogram and (b) segmentation of a myelinated axon that did not show any damage. (c) Tomogram and (d) segmentation of a myelinated axon that showed extensive damage. The black boxed in (a) and (b) indicate the segmented area shown in (c) and (d). Swellings within the myelin sheaths are clearly visible in the highly damaged example. The preserved myelin sheath shows repeated and conserved periodicity of the myelin sheaths. Scale bars a,b: 200 nm, c,d: 50 nm.

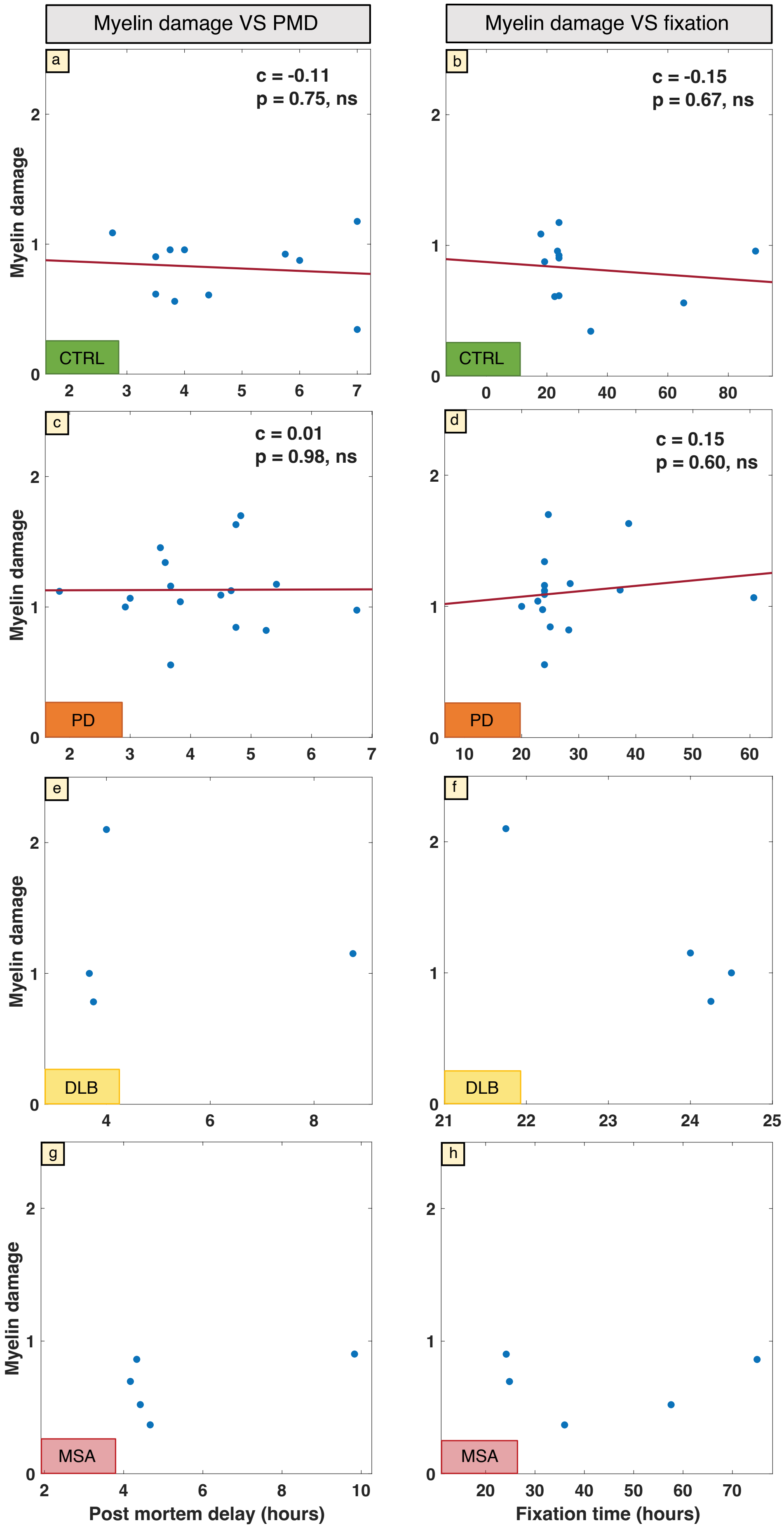
Myelin damage VS PMD
Myelin damage VS fixation
a
c = -0.11
p = 0.75, ns
CTRL
b
c = -0.15
p = 0.67, ns
CTRL
c
c = 0.01
p = 0.98, ns
PD
d
c = 0.15
p = 0.60, ns
PD
e
DLB
f
DLB
g
MSA
h
MSA
Myelin damage
Post mortem delay (hours)
Fixation time (hours)

Supplementary Figure 4: The effect of post-mortem delay (PMD) and fixation time on the presence of myelin damage within the donor groups. Correlation plots of total myelin damage score as a function of PMD (a-c) and fixation time (b-d) for controls and PD. We did not find any significant correlation between myelin damage and PMD or fixation in our study in the single groups, suggesting they may not be the leading cause of the observed differences in myelin damage. The DLB and MSA groups (e-h) were excluded from the statistical analysis due to the small sample size (N=4 and N=5, respectively). c = Spearman correlation coefficient, ns = non-significant.

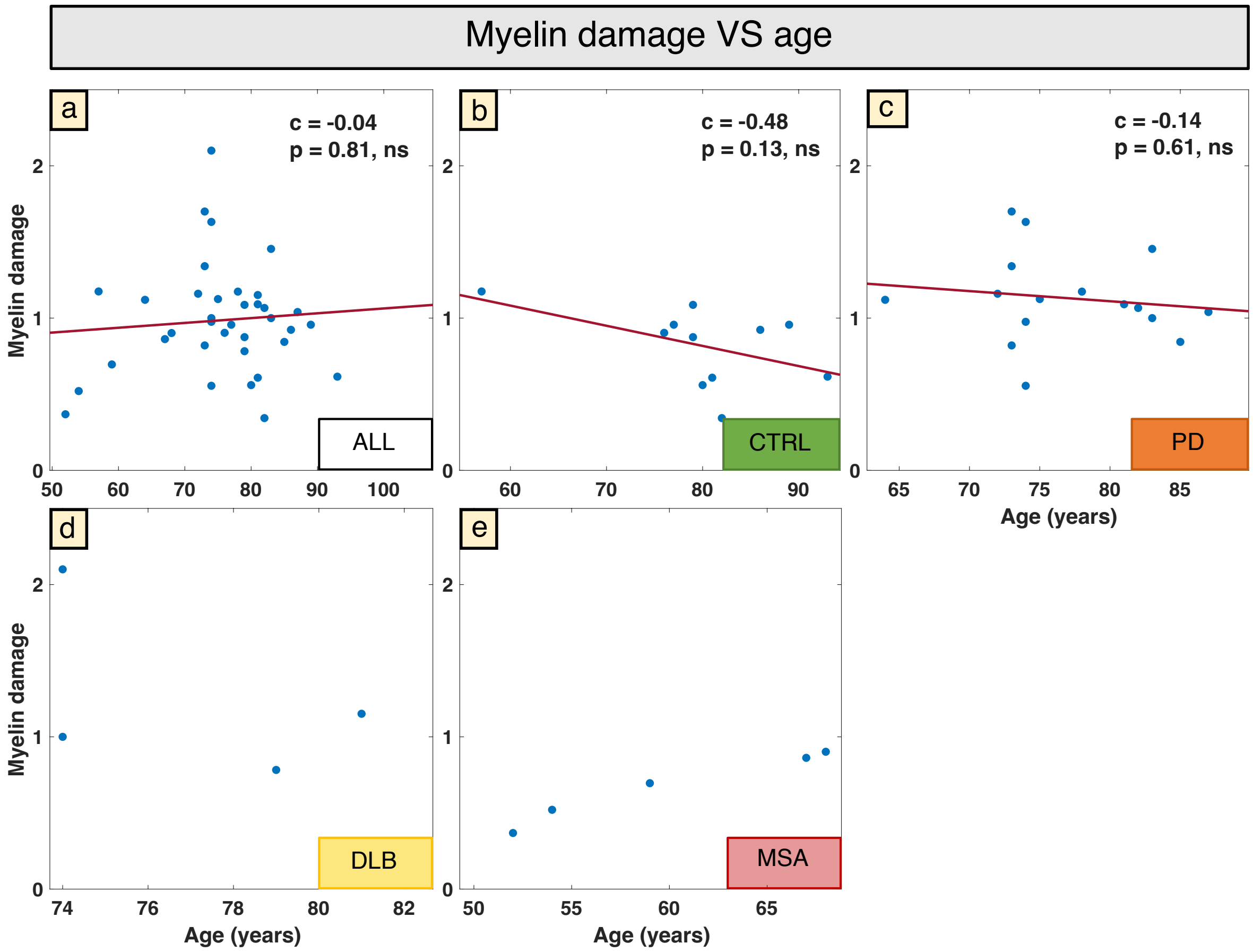

Supplementary Figure 5: The effect of age at death on the presence of myelin damage in the whole cohort and within the donor groups. Correlation plots of total myelin damage score as a function of age in the full cohort (a) and in the groups: CTRL (b), PD (c), DLB (d) and MSA (e). No significant correlation between myelin damage and age at death was observed for any group except MSA (c=0.98, p=0.004). However, due to the small sample size in the MSA group (N=5), this correlation should be interpreted with caution, as small samples can produce inflated correlation coefficients. Therefore, both the DLB (N=4) and MSA groups were excluded from analysis. Overall, the level of damage in the MSA and control groups is comparable, despite age differences, while PD and control groups showed differing levels of damage despite comparable age. These factors suggest that age might not be the leading cause of the observed differences in myelin damage. c = Spearman correlation coefficient, ns = non-significant.

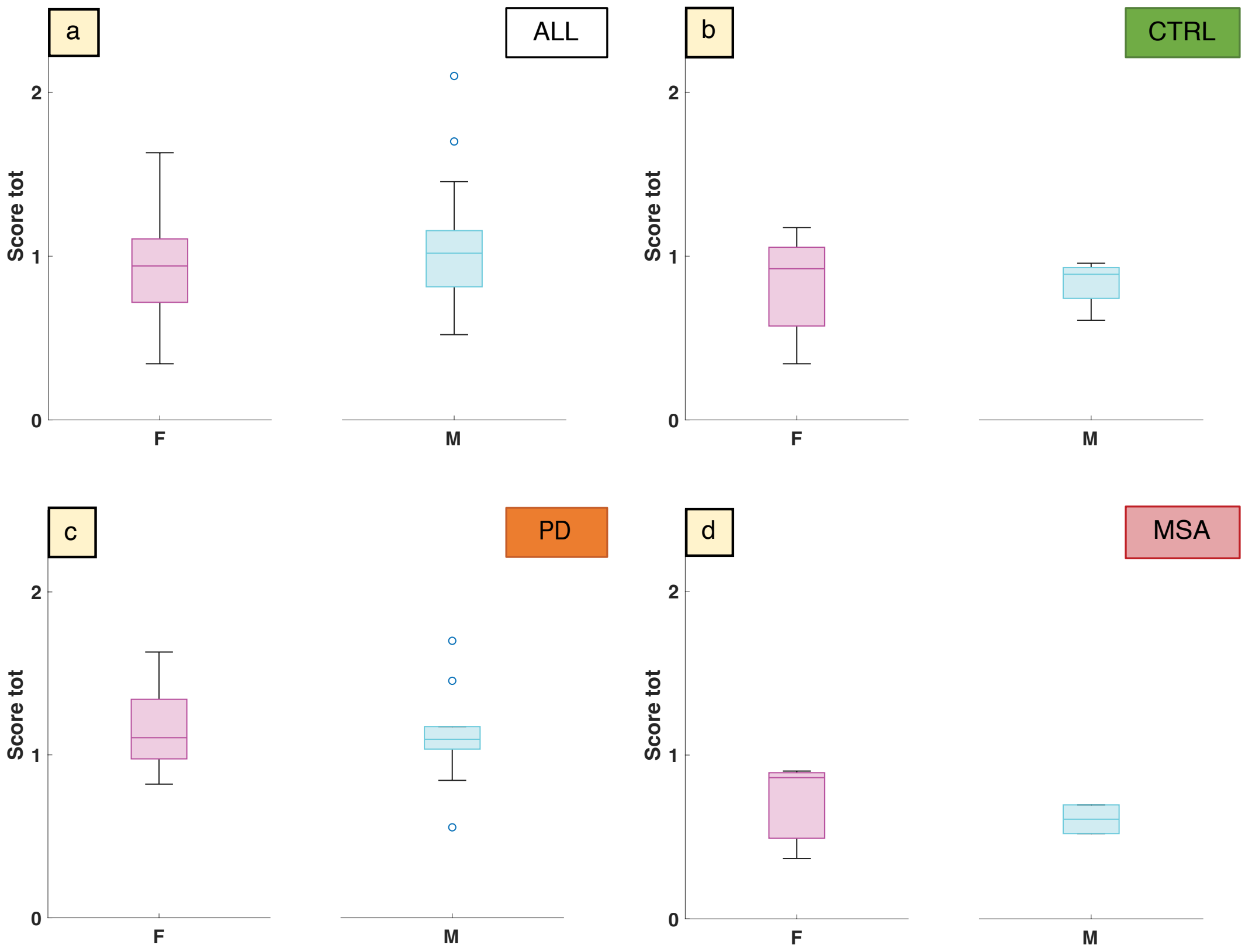

Supplementary Figure 6: Myelin damage does not show sex differences. None of the groups show sex differences in terms of myelin damage as all the p-values are non-significant. Total myelin damage score (Score tot) shown for female (pink) and male (blue) groups. (a) full cohort, (b) control, (c) PD and (d) MSA groups. DLB group not shown as no females were included in the EM study.

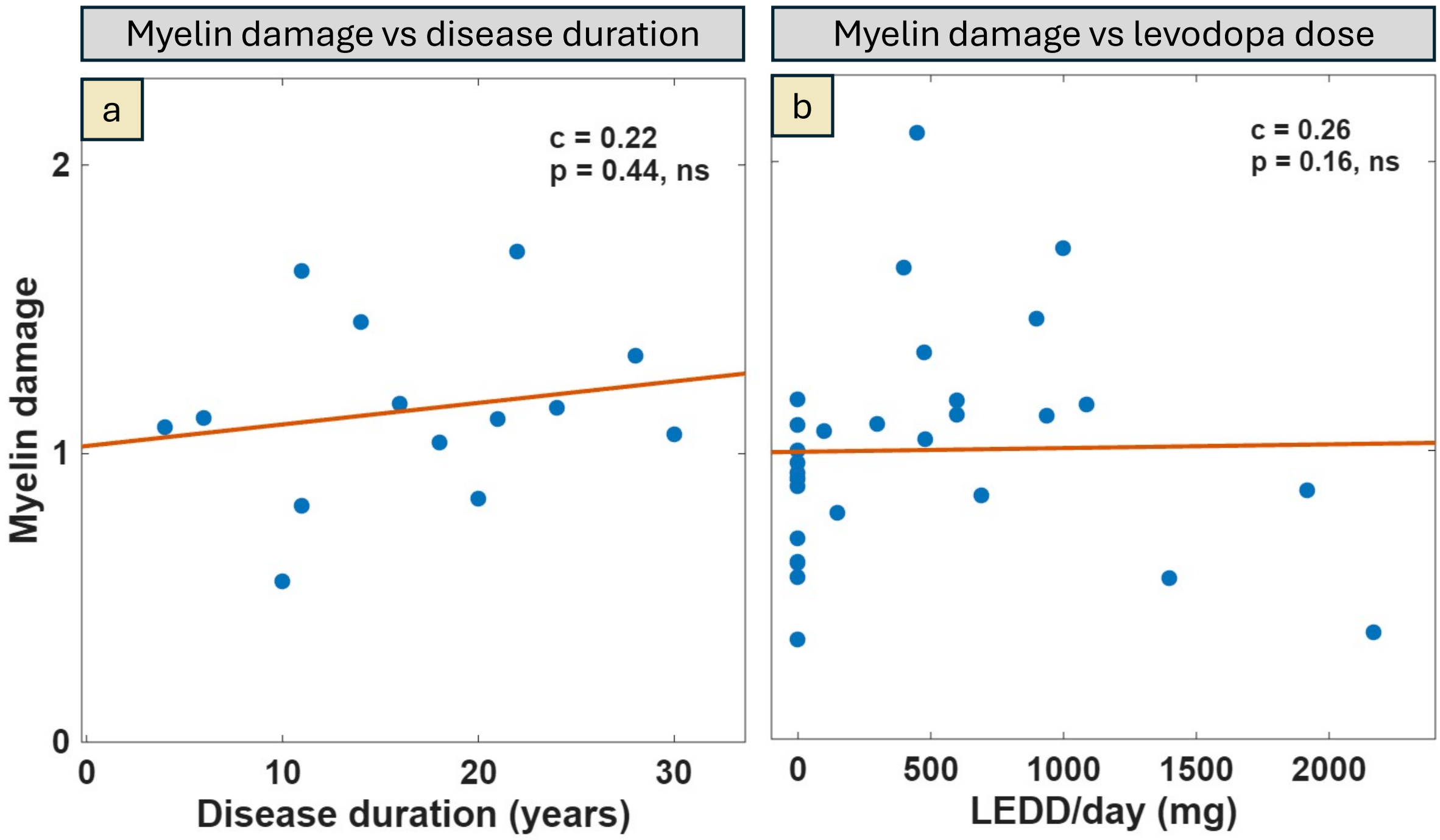

Supplementary Figure 7: Myelin damage does not show any correlation with disease duration in PD (a) and with levodopa equivalent daily dose (LEDD) in the whole cohort (b) as none of the p-values show statistical significance (p>0.05). c = Spearman correlation coefficient, ns = non-significant.

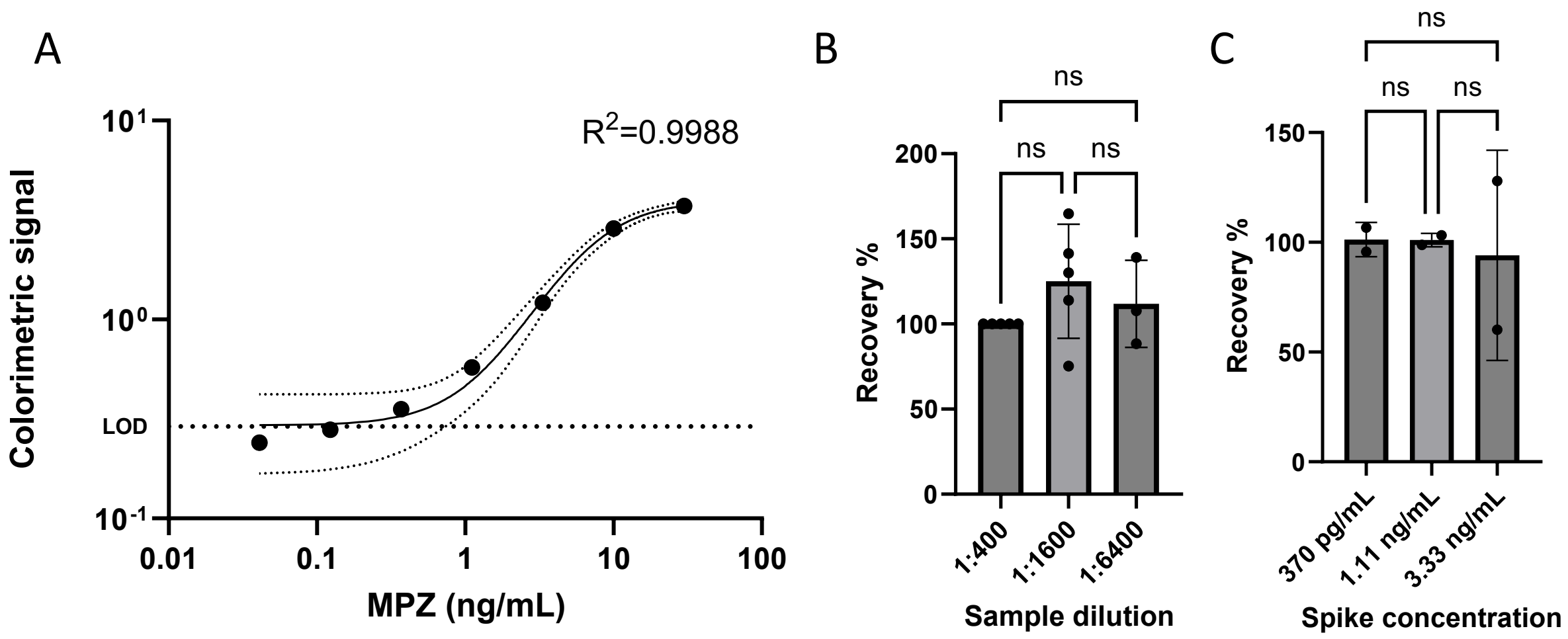

Supplementary Figure 8. MPZ ELISA validation. In-house produced indirect MPZ ELISA had a limit of detection of 184 pg/mL, determined by back calculation of the blank plus three times the standard deviation of the blank based on the standard curve (A). Dilution linearity was confirmed by going from 1:400 (set at 100% recovery) to 1:1600 (112% recovery) and 1:6400 (125% recovery) dilutions with no significant differences between any of the dilutions. For the 1:6400 dilution, 2 out of 5 cases fell below the LOD (B). The mean percentage recovery for the spiking was 101% for the low (370 pg/mL) and moderate (1.11 ng/mL) spiking

conditions, and 94% recovery for the high (3.33 ng/mL) spiking condition (C) There were no significant differences between any of the conditions, confirming the validity of the assay.

Supplementary references

1. Hyman, B. T. *et al.* National Institute on Aging-Alzheimer's Association guidelines for the neuropathologic assessment of Alzheimer's disease. *Alzheimers Dement. J. Alzheimers Assoc.* **8**, 1–13 (2012).
2. Montine, T. J. *et al.* National Institute on Aging-Alzheimer's Association guidelines for the neuropathologic assessment of Alzheimer's disease: a practical approach. *Acta Neuropathol. (Berl.)* **123**, 1–11 (2012).
3. Sun, Y. *et al.* Propofol's effect on the sciatic nerve: Harmful or protective? *Neural Regen. Res.* **8**, 2520–2530 (2013).
4. Zhang, J. & Li, Y. Propofol-Induced Developmental Neurotoxicity: From Mechanisms to Therapeutic Strategies. *ACS Chem. Neurosci.* **14**, 1017–1032 (2023).
5. Farooq, K. & Hunter, J. M. Neuromuscular blocking and reversal agents. *Anaesth. Intensive Care Med.* **9**, 240–243 (2008).
6. Clar, D. T. & Liu, M. Nondepolarizing Neuromuscular Blockers. in *StatPearls* (StatPearls Publishing, Treasure Island (FL), 2026).
